# A Shifty Target: Tumor-Initiating Cells and Their Metabolism

**DOI:** 10.3390/ijms20215370

**Published:** 2019-10-28

**Authors:** Nicole Bezuidenhout, Maria Shoshan

**Affiliations:** Department of Oncology-Pathology, Karolinska Institute, 171 64 Stockholm, Sweden; nicole.bezuidenhout@ki.se

**Keywords:** cancer, tumor-initiating cells, stem cell markers, cellular metabolism, mitochondria

## Abstract

Tumor-initiating cells (TICs), or cancer stem cells, constitute highly chemoresistant, asymmetrically dividing, and tumor-initiating populations in cancer and are thought to play a key role in metastatic and chemoresistant disease. Tumor-initiating cells are isolated from cell lines and clinical samples based on features such as sphere formation in stem cell medium and expression of TIC markers, typically a set of outer membrane proteins and certain transcription factors. Although both bulk tumor cells and TICs show an adaptive metabolic plasticity, TIC metabolism is thought to differ and likely in a tumor-specific and growth condition-dependent pattern. In the context of some common solid tumor diseases, we here review reports on how TIC isolation methods and markers associate with metabolic features, with some focus on oxidative metabolism, including fatty acid and lipid metabolism. These have emerged as significant factors in TIC phenotypes, and in tumor biology as a whole. Other sections address mitochondrial biogenesis and dynamics in TICs, and the influence of the tumor microenvironment. Further elucidation of the complex biology of TICs and their metabolism will require advanced methodologies.

## 1. Introduction

The concept of cancer stem cells (CSCs) as highly chemoresistant, asymmetrically dividing (self-renewing), and tumor-initiating population(s) within a tumor is now well established. Although it is unclear if the term tumor-initiating cells (TICs) is synonymous or not, it is often used to emphasize their role in metastatic disease. Historically, it is possible that the analogy implied in the term “stem cell” did, for some time, limit our view of the TIC phenotype to cells expressing certain stem cell markers and with a glycolytic metabolism, due to the hypoxic environment, and whose mitochondria are few and immature with few cristae and no or little oxidative phosphorylation (OXPHOS). However, from the abundance of reviews in the last few years on the subject of TIC metabolism, it is now clear that TICs are chameleons whose metabolic plasticity appears to support their survival under different conditions of nutrition and other microenvironmental influences, including the various in vitro conditions used for studying them [1,2]. With metabolic plasticity and adaptability being a major challenge to targeting TICs [1,2], it is logical to examine their mitochondrial content and functions.

Here, we will use the terms TIC and TIC-ness analogously with “stemness” and restrict our discussion mainly to studies on carcinoma TICs and a few on glioma TICs. The selected studies have identified TICs based on expression of at least two acknowledged TIC markers and/or in vitro sphere formation. Thus, we have not included studies on cancer cells described only as being metastatic or circulating. After an overview of methods to identify TICs, we review reports on how TIC markers, metabolism and phenotype(s) are associated with one subsection devoted to mitochondrial physiology and one on the need to further consider the microenvironment as a modulator of TIC-ness. In the concluding remarks, we express the hope that advanced technologies will be able to define the hitherto rather shifty features of TIC metabolism.

## 2. Identifying and Isolating TICs

In order to identify and isolate TICs, fluorescence-based cell sorting of suspensions of cell lines or tumor samples was earlier based on extrusion of the dye Hoechst 33342; the extruding cells were named the side population. This method turned out to be less specific than cell sorting based on the expression of specific cell surface markers. Stem cell surface markers that work across several types of solid tumors include Cluster of differentiation 133 (CD133), CD117, and CD44, and for carcinomas also EpCAM, which, however, is often upregulated in, but not restricted to, TICs. CD44 is represented in a host of studies but may pose problems regarding isolation as well as in the interpretation of results, since it presents with several isoforms and cleaved forms with different properties. For example, a recent report shows that the cleaved form CD44ICD promotes TIC-ness [3]. The intracellular alcohol dehydrogenase isoform ALDH1A, often identified and quantified by immunohistochemistry or by measuring enzyme activity using ALDEFLUOR^TM^ staining, was early on identified as a TIC marker in, for instance, ovarian and breast cancer [4,5] and in several other cancers. The G-protein coupled receptor LGR5 is a TIC marker for colon and ovarian cancer [6,7] and glioma [8]. Co-expression of LGR5 and ALDH1 was strongly predictive of metastasis and poor prognosis in non-small cell lung cancer [9]. Expression of certain transcription factors, most of which regulate embryonic stem cells and developmental pathways, is also used for identifying or at least characterizing TICs, notably Nanog, OCT4, SOX2, Bmi1, MYC, Klf4, and Yin Yang-1 (YY1). They may show both nuclear and cytoplasmic localization likely as part of regulatory processes affecting their activity and effects [10].

Sphere formation in vitro under non-adherent conditions and resulting in, for example, mammospheres or neurospheres, is another common method for isolating TICs from cell lines as well as tumor biopsies. Subsequent analysis of the expression of stem cell markers, by cell sorting or gene expression analysis, is not always shown in reports. If the purpose is indeed to obtain TICs, stem cell medium (most often serum-free DMEM/F12 medium supplemented with bFGF, EGF, insulin, and bovine serum albumin) should be used, and the expression analysis is paramount, since many samples and cell lines can be made to form spheres, using, for instance, the hanging-drop method [11] without necessarily being enriched for TICs.

Although not always done, validation of TIC-ness is assessed using the sample’s tumor formation efficiency in xenotransplantation assays by titrating the number of injected cells needed to form tumors (limiting dilution analysis). As a sophisticated next step, genetic lineage tracing assesses TIC-ness in xenografts and organoids and involves labeling the purported TICs with a reporter gene in order to follow its expression in daughter cells and clones over time. This way, clonal development and hierarchical cell clusters can be identified.

### 2.1. Different Types of TICs?

The self-renewal of TICs through asymmetrical division gives rise to bulk tumor cells, but there are also reports of a bi-directional plasticity, i.e., that bulk tumor cells can, under certain as yet undefined conditions, convert or retro-differentiate into TICs [12,13]. A cancer-specific and epigenetically regulated metabolic plasticity might be involved in view of the de-differentiation effects of serum deprivation and some level of nutrient stress, as in stem cell medium, but also in what is often called the cancer stem cell niche (CSCN). Extensive epigenetic regulation in TICs through acetylation would be supported by a good supply of acetyl-CoA from fatty acid oxidation (FAO). A variant on the theme is the yin–yang model which holds that bulk tumor cells and TICs represent proliferating and non-proliferating populations, respectively. This was to some degree tested by Ning et al. [14] who found that when pancreatic cancer cell lines were cultured in stem cell medium, they self-aggregated into spheres within hours and after 5 days expressed Oct-4 and higher Wnt/β-catenin pathway activity than parallel standard-culture cells. Sphere cells were in G1 phase, and when re-seeded in 2D culture, did not express Ki-67, proliferated slowly, and were more resistant to chemotherapy drugs. Finally, the sphere cells, but not standard-culture cells, had tumor-initiating ability in mice. The authors concluded that the role of culture conditions is consistent with the yin–yang model of TIC-ness [14].

Obviously, non-proliferating cells would be resistant to most standard chemotherapies. Kuo and Ann [15] discuss this in terms of chemoresistant subpopulations they called drug-tolerant persisters (DTPs), which have TIC-like phenotypes suggested to be due to the epigenetic signaling that is associated with metabolic reprogramming rather than mutations. In addition to TICs and DTPs, there is another “class” of progression-related cells called metastasis-initiating cells (MICs) possibly with many TIC-like characteristics. The CD36/CD44 oral carcinoma cells mentioned in Reference [16] are an example, as the authors consistently call them MICs. The same study reported that the CD36+ cells showed a weaker EMT expression profile than the CD36-negative counterparts, and the authors suggest that EMT might not always be required for metastasis [16]. It will be interesting to see if this might validate a distinction between MIC and TIC. The question is, whether and how MICs differ from TICs and/or from cancer cells that have simply undergone EMT, a process that shares many, but not all, molecular factors and pathways with TICs.

### 2.2. Different Populations of TICs in a Sample?

Although the studies described below did not specifically study metabolism, they illustrate the question of which and how many TIC markers to use for sorting, and whether such sorting is representative of the whole TIC population as well as TIC properties. Obviously, this poses a problem regarding which selected subpopulation to study for a given set of traits, e.g., metabolism.

We have reported that malignant ascites from ovarian cancer patients contained syncytium-like spheres as well as discrete cells that formed monolayers in culture, and that the material represented at least two types of potential TIC populations—one with Nanog/EpCAM, seen both in the spheres as well as in the monolayer-forming samples, and one with CD44/Oct-4A, found only in the latter [17]. One may also ask which combination of TIC markers results in which phenotype, as for instance in the study by Leng et al. [6]. Using cell sorting, colorectal cancer cells representing five combinations of high/low levels of the markers LGR5, CD44, and EpCAM were isolated and examined for TIC properties and expression of EMT/TIC-related genes. The most pronounced TIC traits were seen in cells positive for all three markers, and, interestingly, only LGR5-positive cells were tumorigenic [6]. A data-mining study found that different tumor types showed different combinations of TIC-associated transcription factors [18]. For example, prostate, lung, and glioma cancers were YY1^low^/SOX2^high^/BMI^high^/OCT4^high^, whereas colorectal cancers and melanomas were YY1^high^/SOX2^high^/BMI^low^/OCT4^high^. Indeed, multidimensional, large-scale approaches to assess co-expression of several TIC markers are being developed. Recently, Erhart et al. [19] used multicolor flow cytometry to simultaneously assess nine glioma TIC markers in seven types of gliomaspheres from cell lines as well as patients. The results showed that out of the nine, the four markers CD44/CD133/ITGA6/CD36 were consistently found in all samples. This or a similar combination would, in light of the tumor-type specific transcription factor profiles above [18], be interesting to examine in other types of cancer. Moreover, it may be a problem or a possibility that specific markers do not necessarily associate with specific properties. Thus, in breast cancer cells, Li et al. [20] showed a direct correlation between the CD44/CD24 ratio and ALDH1 and also showed that, while ALDH1 associated with migration and metastasis, the CD44/CD24 ratio related to proliferation and tumorigenesis.

In summary, to define and isolate TICs, tumor-specific profiles of markers including surface proteins as well as transcription factors may be necessary; similarly, a range of TIC markers may need to be examined in order to examine the regulation of certain TIC properties.

## 3. TIC Markers, Metabolism, and Mitochondria

### 3.1. Background

Historically, normal stem cells and TICs have been described as similar in terms of low proliferation, a hypoxic environment, and harboring small, immature mitochondria, resulting in a glycolytic metabolism rather than one depending on oxidative phosphorylation (OXPHOS) for energy. Accordingly, a recent study showed that glioma TICs under differentiating conditions in the presence of serum went from small mitochondria with few cristae and higher expression of glycolysis-related genes and TIC markers, to well-developed mitochondria and lower expression of glucose metabolism genes [21].

However, the current view is that both non-TIC tumor cells and TICs critically depend on highly functional mitochondria, e.g., a functional tricarboxylic acid (TCA) cycle and fatty acid (FA) β-oxidation; moreover, both categories of cells show a high capacity for adaptation of their metabolism to oncogenic and microenvironmental cues and using a number of different substrates and catabolic pathways [1,22,23]. The high glucose uptake and dependence often seen in non-TIC (bulk) tumor cells provides precursors and reducing equivalents for macromolecule synthesis. Similarly, bulk tumor cells are able to upregulate glutamine metabolism. Glutamine is the most abundant amino acid in plasma; it is taken up by specific transporters and converted by glutaminase into glutamate which is then converted to α-ketoglutarate, an intermediate in the TCA cycle. Glutamine is thus important for energy homeostasis and is a precursor for amino acid and fatty acid (FA) syntheses, the latter via carboxylation of α-ketoglutarate to citrate. In TICs, however, glutamine metabolism is less studied. Altogether, these points raise questions regarding how and to what degree bulk tumor cells and TICs differ with respect to metabolism, and also to what extent and how TIC markers influence metabolism or vice versa. Table 1 is an overview of the references in Section 3.2. below and of associations between specific TIC markers and metabolic phenotypes that they present.

### 3.2. TIC Markers and Metabolism

Exactly how the standard TIC expression markers regulate or influence TIC metabolism remains largely unclear. Most likely, standard TIC markers, such as CD44, ALDH1, CD117, CD133, and EpCAM, influence TIC-ness and TIC metabolism not directly but by affecting certain core signaling pathways that, in turn, regulate the expression and activities of numerous proteins. For instance, ALDH1 is involved in the detoxification of aldehydes caused by reactive oxygen species (ROS) [24] and in the conversion of retinaldehyde to retinoic acid which, via the retinoic acid receptor family, regulates developmental processes, differentiation, and metabolism [25]. These mechanisms might be speculated to play some role in TICs. More well-studied pathways include the Wnt/β-catenin, Notch, Hedgehog, AKT, and STAT3 pathways, as well as SREBP1—a master transcriptional regulator of glucose and lipid metabolism—and AKT/mTOR pathways and MYC, reviewed in References [26] and [27]. However, these and additional pathways relating to metabolic reprogramming, via, for example, Wnt and HIF1α-, are important also in non-TIC tumor cells, and include pathways associated with epithelial–mesenchymal transition (EMT). The latter is believed to be a prerequisite for metastasis and for development of a TIC phenotype, both likely involving metabolic reprogramming [28]. It is thus not clear to what extent EMT signaling overlaps TIC development and maintenance. In addition, several transcription factors display a similar overlap between EMT, dedifferentiation, and TIC signaling, e.g., Snail, STAT3, Nanog, and Oct4. The preeminence of the latter two in stem cell pluripotency and self-renewal has given them weight as TIC markers.

#### 3.2.1. OXPHOS and Glutamine Metabolism in TICs

Counter to earlier models, several recent reports indicate that TICs may depend on OXPHOS rather than glycolysis. To assess OXPHOS dependence, inhibitors of the electron transport chain (e.g., rotenone, antimycin A, oligomycin, and the diabetic drug metformin) are often used. The results suggest that targeting OXPHOS is of interest for eradicating TICs, leading to the development of various potential small-molecule drugs. For example, synthetic small-molecule inhibitors of ubiquinol-cytochrome *c* reductase binding protein (UQCRB) in Complex III blocked neurosphere formation in two glioma cell lines, and OXPHOS inhibitor treatment of neurospheres led to the downregulation of c-Met, STAT3, Akt, and TIC markers CD133, OCT4, Nanog, and SOX2 [29]. A discussion on the use of OXPHOS inhibitors for therapeutic purposes is presented in Reference [30].

In one study, antimycin A blocked sphere formation of the side population of lung cancer cells and decreased the expression of CD133, Nanog, and SOX2 as well as β-catenin [31]. Another early demonstration of TIC dependence on OXPHOS is found in a study by Pastò et al. [32] who used ascitic effusion cells from ovarian cancer patients to show that cell sorting based on CD44 and CD117 yielded cells with sphere-forming ability as well as higher expression of Nanog, SOX2, OCT4, ALDH1A, and the EMT regulators Snail2 and TWIST1. In vitro, these CD44^+^/CD117^+^ TICs showed higher ROS levels and sensitivity to antimycin A and several other inhibitors of OXPHOS, compared to CD44^+^/CD117^-^ populations; moreover, upregulation of enzymes involved in OXPHOS, TCA cycle, pentose phosphate pathway (PPP), and fatty acid oxidation. While the CD44^+^/CD117^+^ TICs, but not the CD44^+^/CD117^-^ cells, survived glucose starvation with intact OXPHOS, their uptake of glucose in full medium was taken to reflect the need for glucose-fueled pentose phosphate pathway (PPP) activity to provide NADPH as a modulator of redox homeostasis in the face of the high OXPHOS activity. Altogether, the presence of CD117 thus reflected some form of metabolic benefit.

As part of their demonstration that MYC and the Bcl-2 family protein MCL1 induce OXPHOS-dependent TIC-ness, Lee et al. [33] found that the ALDH^+^/CD44^+^/mammosphere TIC fraction of triple-negative breast cancer cells showed increased mitochondrial membrane potential and respiratory capacity, and conversely, that cells with these properties formed mammospheres in an oligomycin-dependent manner. The same study also showed that siRNA-mediated downregulation of MCL1 led to reduced levels of TCA cycle intermediates, suggesting that the supportive role of MCL1 in TIC-ness involves increased oxidation of mitochondrial fuels [33]. In line with this, a study on ALDH1-positive and negative xenografts identified an ALDH1-selected 19-gene core signature of breast cancer TICs that included genes involved in OXPHOS, lipid metabolism, cell cycle regulation and detoxification [5]. Importantly, MYC-driven upregulation of OXPHOS dependency was reported also by Sancho et al. [34], in CD133^+^ pancreatic cancer TICs, along with sensitivity to metformin which is regarded as a Complex I inhibitor. The upregulation was found to depend on MYC-driven upregulation of PPARgamma co-activator 1α (PGC1α) [34], a transcriptional co-factor with a major role in regulation of mitobiogenesis and mitochondrial function [35].

Regarding CD44, its overexpression has been shown to upregulate the glycolysis enzyme PFKFB4 [36,37], and in line with this, CD44 knock-down in breast cancer cell lines led to reduced glycolysis and AKT activity [38]. In one out of two pancreatic cancer cell lines, the drug dichloroacetate (DCA), which by inhibiting pyruvate dehydrogenase kinase (PDK) stimulates OXPHOS and a concomitant decrease in glycolysis, reduced the CD44/EpCAM-expressing population, but inhibited sphere formation in both [39]. This indicates a context-dependent role of CD44 and metabolism. However, the various CD44 isoforms may need more investigation in regard to metabolism, since a recent report shows that the shortest isoform, CD44s, inhibits and that the intracellular, cleaved form CD44ICD promotes TIC-ness [3].

An association between CD133 and OXPHOS was reported by Denise et al. [40] who showed enhanced sphere formation and CD133 expression in colon cancer cell lines made resistant to 5-fluorouracil, along with increased OXPHOS activity and high ROS, reduced glucose uptake and PPP activity, and a small effect of metformin on proliferation. Interestingly, acute re-treatment of the resistant cells with 5-fluorouracil induced a massive increase in CD133, and sphere formation that were all but eradicated by OXPHOS inhibitors (including metformin) but insensitive to the glycolysis inhibitor 2-deoxyglucose [40].

That metformin has been shown in a number of reports to target and even eliminate TICs, especially in combination with chemotherapy, is often taken as evidence that they are OXPHOS dependent, although it affects also other pathways such as AMPK. Metformin treatment selectively reduced the CD44^+^/CD133^+^ fractions in four out of eight colorectal cancer cell lines and prevented sphere formation in said population [41]. The inhibitory effect involved activation of AMPK, a known metformin target, but also OXPHOS. Assays of cells cultured under different nutrient conditions indicated that the metformin resistance of the TIC fraction in one cell line was due to glutamine-mediated compensation. Accordingly, combining metformin with a glutaminase inhibitor had a sensitizing effect both in vitro and in xenografts, and knock-down of glutamine metabolism enzymes significantly decreased the CD44^+^/CD133^+^ fraction in the metformin-resistant cells [41]. On the same note of glutamine-supported TICness, sphere formation of head-and-neck cancer cells were found to require glutamine, and the spheres showed higher levels of glutaminase expression and glutamate than non-sphere forming cells [42]. When sorted CD44/ALDH1-expressing cells were further studied, chemical inhibition and knockdown of glutaminase suppressed CD44 and ALDH1 expression, respectively [42].

#### 3.2.2. Fatty Acid Metabolism in TICs

There has recently been a great increase in interest in fatty acid (FA) metabolism in TICs, both as a defining and supporting process as well as a promising target for therapy. TICs often show upregulated FA metabolism [27], although it is not always clear whether from a low background or an already upregulated level. Breast cancer TICs with the canonical CD44+/CD24-/EpCAM+ signature showed massive upregulation of FA biosynthesis enzymes FASN, ACLY, ACC1 and the master regulator SREBP1 [43]. In a glioma model, FASN expression and lipid synthesis were high in patient-derived TICs, along with glioma TIC markers SOX2, fatty acid binding protein-2 (FABP2) and nestin, and FASN inhibition led to downregulation of these markers as well as decreased viability and invasivity [44]. Another study compared monolayer and sphere cultures of a pancreatic cancer cell line, in standard serum-containing cell culture medium and stem cell medium, respectively [45]. Although the study did not report TIC marker expression, the purported TIC spheres were found to upregulate FASN and enzymes in the mevalonate pathway; accordingly, inhibition of FASN or the mevalonate pathway, using cerulenin or atorvastatin, respectively, led to reduced viability in the TICs but not in the monolayer cells [45]. The authors also pointed out that studies on statins used as cholesterol-reducing drugs have shown a potential cancer-protective effect. Moreover, glycolysis enzymes were upregulated, and TCA cycle enzymes were downregulated in the TICs, suggesting downregulation of OXPHOS. Using metformin might have indicated whether they were indeed less OXPHOS dependent than the monolayer cells.

In addition to FA biosynthesis, increased uptake via the membrane transporter CD36 may provide the cell with FA. While not normally expressed in the brain, CD36 is a TIC marker in glioma, in which it promotes TIC-ness and drives progression [46]. The combined expression of CD36/CD44/CD133/ITGA6 was found to be a signature of glioma neurospheres from different sources and to be a marker of worse prognosis in patients [19]. In oral carcinoma cell lines and patient samples, CD36^+^/CD44^+^ cells were slow-cycling, and, in accordance with transcriptome signatures representing lymph node metastasis, they also homed to lymph nodes in xenografts [16]. That this homing was blocked by anti-CD36 blocking antibodies and boosted by the FA palmitic acid or a high-fat diet suggests that the role of CD36 was indeed to provide FA rather than its other roles as a collagen I and thrombospondin receptor. The authors point out that CD36 predicts poor prognosis in several types of cancer [16]. We have observed upregulation of CD36 in SKOV-3-R, a cisplatin-selected subline of SKOV-3 ovarian cancer cells, a multi-drug resistant subline and which, unlike the parental line, forms spheres in stem cell medium and expresses ALDH1A, CD117 and CD44 [47]. Regarding FA uptake, the roles in cancer and TICs of fatty acid transporter protein (FATP) family of transporters may need to be more studied than is the case at present.

The fatty acids—whether synthesized or imported—are used by cancer cells either for catabolism, usually via energetically efficient FA β-oxidation (FAO) in the mitochondria, or stored as lipids in lipid droplets (LDs), or as precursors or phospholipids which may, per se, be bioactive, such as mitogenic lysophosphatidic acid (LPA), or incorporated into membranes.

For TICs and bulk cancer cells alike, the benefits of upregulated FAO include lower ROS levels per ATP, and high levels of the end-product acetyl-CoA needed for acetylation-based epigenetic regulation. The carnitine palmitoyl transferase (CPT1) is required for FA transport into mitochondria, wherefore isoforms of this enzyme are being investigated as therapeutic targets. Inhibitors and knock-down strategies showed that the JAK/STAT3 pathway regulates CPT1 and FAO, and thereby self-renewal in breast cancer CD44^+^/CD24^−^ mammosphere TICs [48]. Mechanistically, STAT3 knockdown led to decreased expression specifically of CPT1B. Addition of acetyl-CoA or the fatty acid myristic acid, which unlike the physiologically more relevant palmitic or oleic acids bypasses CPT1, reversed the effects of STAT3 knockdown, confirming the role of FAO in self-renewal [48].

Neutral lipids formed from FAs may accumulate in lipid droplets (LD) which are sometimes described as “lipid sinks” that protect cells against lipotoxicity, and sometimes as a safeguard under conditions of nutrient depletion. In bulk tumor cells, the LD content is often higher than in normal tissue and correlates with worse prognosis. Research on LDs in cancer cells is exemplified in a paper by Jarc et al. [49] showing how LDs in breast cancer cells coordinate uptake and release of, in particular unsaturated FAs, and that these have an antioxidant as well as nutrient stress protective role. However, the role of LDs in TICs is largely unexamined. The previously mentioned study on CD36^+^/CD44^+^ cells [16] showed that CD36-depleted cells developed LDs and showed reduced metastatic capacity. Similarly, STAT3 knockdown in breast cancer TICs described above induced accumulation of LDs [48]. Further research questions thus include whether LD content is generally higher in bulk tumor cells than in TICs, despite the correlation with worse prognosis, and whether this could due to a much higher turnover of FAs and lipids in TICs, and/or linked to differential expression of FASN, CD36 and FAO enzymes. This is also related to the question whether contents and functions of LDs change dynamically during cancer progression, as outlined in a review by Tirinato et al. [50]; the review also details the biogenesis and various functions of LDs.

Unsaturated FAs increase membrane fluidity. This property remains unclear in TICs, but increased membrane fluidity is important in migration, mitosis, signal transduction and cell polarity. Mono-unsaturated FAs (MUFA) generated by stearoyl-CoA desaturase-1 (SCD1) appear to be of particular importance and may correlate with ALDH1. This is exemplified by ovarian cancer ALDH1/CD133-sorted cells with significantly higher levels of MUFAs compared to their non-TIC counterparts, and whose viability, sphere formation and in vivo tumorigenicity were suppressed by inhibition of desaturases [51]. Moreover, the TICs were enriched for SCD1, and shRNA knockdown of SCD1 had similar effects as SCD1 inhibitors, including downregulation of SOX2, Nanog, and Oct4. Inhibition of SCD1 also downregulated NFκB-regulated IL-6 expression, and a positive feedback loop connecting NFκB, ALDH1A1, and SCD1 was found [51]. Similarly, a metabolomic study on colorectal TICs showed increased levels of unsaturated FAs and lipids, and in line with Reference [51], inhibition of NFκB, ALDH1A1, and SCD1, respectively, reduced sphere formation [52]. It is interesting to note that in a mouse keratinocyte cell line, inhibition of SCD1 as well as addition of its product oleic acid affected membrane fluidity and thereby Wnt-signaling [53].

Regarding bioactive phospholipids, LPA and sphingosine-1-phosphate (S1P) are involved in regulation of normal stem cells and development [54]. Mitogenic LPA is generated by the enzymatic activity of autotaxin (ATX) on lysophosphatidylcholine, and both LPA and ATX may be secreted as autocrine factors. Autotaxin is upregulated in many cancers, and is regulated via OCT4, SOX2, and Nanog; an association between ATX and ALDH1 has also been observed in ovarian cancer tumorigenic sphere cells [55]. Moreover, the same study shows that ovarian cancer sphere cells from cell lines and primary tumors maintain TIC-ness through an LPA autocrine loop, and that inhibition of ATX-LPA/AKT signaling prevents chemoresistance and tumorigenicity in these cells [55].The lysosphingolipid S1P is released from ceramides by ceramidases and phosphorylated by sphingosine kinase. The CD133-expressing glioma TICs were found to overexpress acid ceramidase (ASAH1), and several inhibitors of this enzyme efficiently induced apoptosis in these TICs [56]. Likewise, in melanoma cells, knockout of ASAH1 induced apoptosis, reduced ALDH1A3 expression and prevented sphere formation [57].

Nanog may be central to FA metabolism in many types of TICs [27]. It supported TIC-ness in hepatocellular carcinoma by upregulating FA oxidation and repressing genes involved in OXPHOS, with concomitant inhibition of oxygen consumption and ROS production, along with increased chemoresistance [58]. Conversely, restoration of OXPHOS and downregulation of FAO reduced tumorigenicity and chemoresistance. In one prostate and two ovarian cancer cell lines, knock-down or inhibition of the mitochondrial pyruvate carrier (MPC) led to increased expression of Nanog, CD44, ALDH1, and HIF1α [59]. Interestingly, knock-down of MPC in crypt base columnar cells of the small intestine expanded the stem cell compartment and induced an FA-dependent metabolism [60]. It would be interesting to assess MPC status and ALDH1 and CD133 expression in for instance breast or ovarian TICs and, conversely, effects of MPC knockdown or ectopic expression of Nanog, under standard as well as oxygen/nutrient deprivation conditions.

Studies on FA and lipid metabolism in TICs have touched on the role of obesity in tumorigenesis and chemoresistance, and vice versa. That obesity-associated inflammation is an important driver in many types of cancer is now accepted, and involves not only macrophage cytokines and the like, but also adipokines, or adipose-tissue derived factors. Leptin is one such adipokine, which acts through JAK2/STAT3 pathways to support TICs by promoting FAO [61]. Leptin levels in serum or ascites predict outcome in several types of gynecological cancers [62]. Compared to lean controls, obese mice with MMTV-Wnt tumors showed lower survival and had higher expression of TIC/EMT-related genes; moreover, serum from obese mice had significantly higher levels of leptin and could increase mammosphere formation and induce TIC-associated expression such as ALDH1A, SOX2, etc. [63].

Finally, gluconeogenesis, or “reverse glycolysis” is seen in cancer cells under nutritional stress. Phosphoenolpyruvate carboxykinase (PEPCK) is involved and converts oxaloacetate to phosphoenolpyruvate. In total, PEPCK and gluconeogenic activity support pyruvate cycling, serine and ribose synthesis, glutamine- or lactate-derived FA and glycerol synthesis in starved cells [64]. As PEPCK/gluconeogenesis has not been investigated in TICs, it might thus be of interest to examine it under different culture conditions, including standard culture, stem cell medium, sphere cultures, and in contexts of FA metabolism and autophagy.

## 4. Mitochondrial Physiology in TICs

Normal and induced pluripotent stem cells (iPSC) and some TICs are reported to be glycolytic and to have few and immature mitochondria which upon differentiation increase in number and development of cristae [65]. However, in accordance with high OXPHOS, FAO, and TCA cycle activity, several recent findings indicate that TICs differ from bulk tumor cells in terms of increased mitochondrial content and physiology. The higher mitochondrial activities provide not only energy and building blocks, but also metabolites that are instrumental to epigenetic alterations that may serve the TIC phenotype, e.g., acetyl-CoA, succinate, S-adenosylmethionine, and others. Recent research in this field is reviewed in [66], where the authors coin the term *mitostemness* to signify the role of mitochondria in TIC self-renewal and resistance to differentiation. The concept also covers mitochondrial dynamics (here briefly overviewed in Section 4.2), mito-nuclear communication and the mitoproteome.

### 4.1. Mitochondrial Biogenesis

Mitochondrial content or mass can be gauged as the ratio of selected mitochondrially encoded genes (at least three are recommended) to a nuclear gene and/or by staining with fluorescent probes, such as MitoTracker Green and nonylacridine orange which are independent of membrane potential; their use is examined in [67]. Levels of certain mitochondrial proteins can also be used, e.g., VDAC.

Based on MitoTracker staining and cell sorting of two breast cancer cell lines, Farnie et al. [68] found that populations with high mitochondrial content were enriched for ALDH1 and could form mammospheres; similarly, patient biopsies showed a correlation between ALDH1 and mitochondrial content. Mitochondrial content was increased also in the 5-FU resistant CD133-positive colon cancer cells in the study by Denise et al. [40], along with higher mitochondrial membrane potential and increased coupling between oxygen consumption and ATP production.

Higher mitochondrial content prompts examination of transcriptional regulators of mitochondrial biogenesis such as PGC1α and MYC. The transcriptional co-activator PGC1α is almost invariably called a master regulator of mitobiogenesis and function, although it has a variety of functions in different tissues, in part due to its partnering with different regulators, notably peroxisome proliferator-activated receptor-gamma (PPARγ) and estrogen-related receptor α (ERRα) [35,69]. Its downstream effectors include the mitochondrial transcription factor M (TFAM) and antioxidant defense factors; this indicates its “double-edged” role in supporting OXPHOS as well as ROS protection. Similarly, PGC1α has been shown to have tumorigenic and tumor suppressive features and to predict worse as well as more favorable outcomes [70]. Thus, the association between PGC1α and OXPHOS in cancer is well-studied, but its functional roles are difficult to generalize, including in TIC metabolism. However, in glioma, expression of PGC1α correlated with worse outcome, and, in vitro, shRNA-mediated knock-down suppressed expression of mitochondrially encoded electron transport chain (ETC) genes as well as TIC features; when xenografted, the resulting cells were also less proliferative and less invasive [71]. XCT790, a specific inhibitor of ERRα, was found to prevent mammosphere formation and TIC marker expression [72]. By contrast, our SKOV-3-R CD44+/CD117+/ALDH1+ cells (see Section 3.2.2.) lack PGC1α expression and show approximately half the PGC1β expression of the parental cells, yet form spheres, show marker expression and a small but significant increase in mitochondrial content [47,73]. In line with PGC1α-independent upregulation of mitochondria, we have reported that in a tissue microarray of ovarian cancer, the high-grade serous carcinomas expressed high levels of PGC1α, whereas in the clear-cell subtype PGC1α and TFAM were undetectable, along with high levels of the mitochondrial marker VDAC [73].

As sphere formation may be stimulated by increased ROS [74], it might trigger PGC1α as an antioxidant response. ROS were indeed found to induce PGC1α expression in ALDH1-enriched spheres from an ovarian cancer cell line, and expression was inhibited by ROS scavengers [75]. In the same study, malignant ascites cells from different histological types of ovarian cancer formed ALDH1-enriched spheres with PGC1α upregulation and increased mitochondrial content [75]. We have earlier mentioned metformin-sensitive PGC1α-expressing pancreatic cancer TICs with increased mitochondrial content [34]. Moreover, metformin resistance developed in a subpopulation which expanded, showing EpCAM/CD44/CD133 expression, upregulation of MYC and downregulation of PGC1α, i.e., the reverse of the original heterogeneous population. Additional experiments indicated that MYC binds to the PGC1α (*PPARGC1A*) promoter and that it may act as an upstream inhibitor of PGC1α [34]. There was thus a switch over time and an inverse correlation between MYC and PGC1α.

Interestingly, MYC was upregulated in ALDH1-enriched mammosphere cells from cell lines and triple-negative breast cancers; this was seen also in CD44-expressing cells [33]. Knockdown of MYC inhibited mammosphere formation and decreased the ALDH1 and CD44-expressing populations. Next, ALDH1/MYC high and low cells were compared in terms of metabolism: ALDH1^+^ cells had higher basal and maximal respiratory capacity and mitochondrial membrane potential (MMP). Conversely, cells with high MMP showed higher mammosphere capability. Experiments with inhibitors, siRNA and ectopic MYC expression showed that MYC was indeed responsible for increased mitobiogenesis and OXPHOS activity and the subsequent enrichment of TICs [33]. Again, to further understand if and how PGC1α and MYC differ with regard to regulating TIC-ness, it would be interesting to see whether PGC1α was at all expressed or affected in these experiments.

### 4.2. Mitochondrial Dynamics

Mitochondria show intracellular organization ranging from filamentous, or tubular, networks of fused mitochondria to fragmented networks in which mitochondrial fission has resulted in individual mitochondria. The underlying processes of fusion and fission are collectively named mitochondrial dynamics, and they regulate the morphology, number, distribution, ROS levels and respiratory capacity of mitochondria [76]. Fusion involves the mitochondrial transmembrane proteins mitofusins 1–2 (Mfn1–2) and Opa1, among others. Fission depends mainly on the cytosolic GTPase Drp1 and its various posttranslational modifications and interacting proteins, e.g., Fis1 and Mff. Drp1 is instrumental in coupling mitochondrial morphology and cell cycle [76]. Mitochondrial dynamics in cancer metabolism are reviewed in more detail in References [77,78].

Compared to non-TIC counterparts, glioma TICs showed fragmented mitochondria (fission) and activated (phosphorylated) Drp1; siRNA- or drug-mediated downregulation of Drp1 induced apoptosis and decreased tumor growth [79]. Similarly, in ovarian cancer ALDH1+ spheres, mitochondrial networks were fragmented and more perinuclear compared to the tubular ones in the non-TIC cells [75]. In prostate cancer TICs, knock-down of the Drp1-interacting protein Mff led to reduced viability and tumorigenicity [80]. The same study also indicated that fission enabled asymmetric division and self-renewal [80], i.e., key aspects of stemness. This is in line with Drp1 being crucial for self-renewal, and with dedifferentiated cells being characterized by mitochondrial fission [65,66]. If and how fission specifically in TICs is associated with proliferation is unclear, but may be cell- or tissue-type dependent, as seen in several studies on normal stem cells and bulk tumor cells [65]. Altogether, we thus expect increased interest in mitochondrial dynamics as a regulator of TIC metabolism and de-/differentiation.

## 5. The Microenvironment

As briefly mentioned also in Section 2.1., culture/growth conditions may affect TIC-ness as well as our definitions of TIC-ness. Hypoxia is a primary aspect to consider. Whether experimental, or in spheres or tissue and the distance to the nearest vessel, it affects metabolism in bulk tumor cells and TICs alike, generally via HIF1α. Hypoxia and HIF2α promoted stemness, seen as neurosphere formation and expression of OCT4, Nanog and MYC, in non-TIC glioma cells [81]. However, contrasting roles of HIF2α, notably in neuroblastoma, have been described [82], suggesting that its roles in TICs be further researched.

In addition to hypoxia, the model presented by Melzer et al. [13] for the CSCN and development of TICs also involves an ecology of tumor cells with endothelial, mesenchymal and immune cells, growth factors, hormones, and the specific ECM composition that to a considerable degree is affected by the actions of a number of cancer-associated proteases. The authors stress a role for exchange of cellular material from other cells in the form of mesenchymal stem cells forming cell hybrids with tumor cells, as well as uptake of exosomes and microvesicles.

The role of the extracellular matrix (ECM) in shaping TIC phenotypes needs to be considered, not least because studies on TIC metabolism are based on in vitro work on dissociated and/or sphere-enriched TICs without much involvement of ECM proteins. Extracellular matrix signaling through integrins may affect metabolism, invasion, chemoresistance, and even such physical properties as ECM stiffness can regulate metabolism—for instance, upregulation of both glycolysis and glutamine metabolism in both tumor cells and carcinoma-related fibroblasts (CAFs) [83]. In normal epithelial cells, loss of cell–cell and ECM contacts leads to cell death, whereas cancer cells are protected. A report on the ECM glycosaminoglycan hyaluronan and CD44-mediated oncogenic signaling in HNSCC TICs [84] illustrates the possible effects of specific ECM components, the levels of which can differ between tumors. Mason et al. [85] discuss how metastasizing cancer cells survive detachment from the ECM and report that survival hinges on upregulated glutamine metabolism and antioxidant enzymes, notably Nrf2.

Importantly, similar observations led Batlle and Clevers [2] to point out that cell samples that are titrated for tumor initiation in mouse models lack the important crosstalk with ECM proteins, wherefore their tumor-initiating efficiency may reflect only a highly efficient adaptation to suspension and non-attachment. Thus, physiological TICs within a carcinoma might well have other functions and features with bearing on chemoresistance and metastasis [2]. This would include the feared ability of TICs to lie dormant, perhaps for years—an ability that is not captured by the mouse model tumor titration assay.

Yet, other factors of the microenvironment can potentially regulate TIC development or maintenance. Although still debated and little studied with regard to TICs, horizontal or cell-to-cell transfer of mitochondria [86,87] can be speculated to support the adaptability and survival mechanisms of TICs and how they in turn affect the tumor microenvironment. Much research effort has been, and is, put into elucidating the roles of stromal cells, such as cancer-associated fibroblasts, adipocytes and macrophages. These can be recruited for the benefit of the tumor cells/TICs, and provide them with, for instance, lactate and fatty acids, and cytokines to trigger or support properties such as angiogenesis and motility [88]. The stromal compartment also contributes to the altered cytokine signaling in cancer. The cytokines IL-8/CXCL8 and IL-6 are both upregulated in many tumors and well-known to promote tumor progression. Regarding TIC-ness, it has been shown that conditioned medium from certain hypoxia-cultured breast cancer tissue and cell lines stimulated mammosphere formation via IL-6 and JAK2/STAT3-signaling [89]. Moreover, IL-8 may affect development of TICs by way of upregulating GLUT3 and glucose uptake, as well as hexosamine biosynthesis leading to increased O-GlcNAcylation which in turn was shown to be important for TIC maintenance and tumor formation in vivo [90]. Connecting to recent research on low-grade inflammation and obesity being a risk factor both for some cancers and for therapeutic resistance, we have already mentioned (in Section 3.2.2.) adipocyte-derived leptin and how it regulates TIC-ness via JAK/STAT3 and FAO [61,62,63]. However, by and large, little is known about how cytokines affect TIC metabolism as such.

## 6. Concluding Remarks

We have here touched upon the problems of isolation protocols and how to define TICs in order to be able to study and understand them. One might indeed ask whether it is at all possible to, even within a specific cancer type or in a given clinical sample, lasso the elusive unicorn and discern TIC-specific metabolic traits that are not also seen in bulk tumor cells, since the latter can also switch between glycolytic and OXPHOS phenotypes as well as show upregulation of FA and glutamine metabolism, and since they likely respond in similar ways to stimuli such as ECM cues, chemokines, hormones etc. However, the observed various overlaps between bulk tumor cells and TICs might reflect a spectrum of pathways and profiles, rather than a dichotomous difference. Using advanced mathematical modeling and experimental validation, Jia et al. [91] identified core signaling responsible for a OXPHOS/FAO/glycolytic hybrid metabolism in bulk cancer cells and which the authors propose explains metabolic plasticity. Similar modeling strategies might prove useful for identifying a TIC-type metabolism at the end of the spectrum. Altogether, we speculate that such a profile will involve a superior ability of TICs to withstand severe nutritional stress and coupled to probably intermittent senescence-like states that contribute to chemoresistance and dormancy.

Other emerging methods and technologies show further promise in, for instance, development of organoid cultures containing stromal cells and ECM proteins and in methodical assessments of the effects of growth conditions and various culture media. Even greater is the promise regarding studying in vivo effects on TIC populations. One example of many is that in order to study the role of PPARα, master regulator of lipid and carbohydrate metabolism, Haynes et al. [92] knocked down PPARα in isolated glioma TICs, and tagged the cells with luciferase which were then xenografted into the striata of mice. Bioluminescence and MRI were then used to demonstrate that in the context of a physiologically complete microenvironment the knock-down TICs, but not controls, showed reduced in vivo proliferation, invasivity and tumorigenicity. A second example is a metabolomic study on colorectal TICs, in which single-probe mass spectrometry was used to identify metabolites and metabolite profiles in very little material (single-cell analysis) [52]. This technique could be useful both for comparing different types of TICs and for characterization of TICs versus non-TICs. On a more theoretical plane, it might also be useful to view the metabolic profiles of adherent cancer cells, migrating EMT cancer cells and TICs as a multidimensional continuum which the ever-increasing development of databases and bioinformatics will help us describe and understand and use for practical therapeutic purposes. Table 2 sums up some areas or topics we believe are of importance for further research and understanding of TICs.

## Figures and Tables

**Table 1:** Overview of references on tumor-initiating cell (TIC) markers and associated metabolism. Numbers indicate references in the reference list which suggest an association between the indicated TIC marker and a metabolic phenotype. Abbreviations in brackets after each reference indicate the type of cancer in the study.

	OXPHOS	Glutamine Metabolism	FA Metabolism	Glycolysis
CD44	32 (ov)	41 (co), 42 (hn)	43 (br), 48 (br)	36 (pr), 37 (pr), 38 (br), 39 (pa), 41 (co)
CD117	32 (ov)			
CD133	29 (gl), 31 (lu)	41 (co)	51 (ov),	40 (co), 41 (co)
ALDH1	5 (br), 33 (br)	42 (hn)	51 (ov), 52 (co), 55 (ov), 63 (br)	
MYC	5 (br), 33 (br), 34 (pa)			
Nanog	29 (gl), 31 (lu), 58 (hep) *		58 (hep)	59 (pr)
Oct4	29 (gl)			

Abbreviations: br: breast cancer; co: colon/colorectal cancer; hep: hepatic cancer; hn: head and neck cancer; gl: glioma; lu: lung cancer; ov: ovarian cancer; pa: pancreatic cancer; pr: prostate cancer. * inverse relationship.

**Table 2:** Some key topics in further research on TICs. Numbers refer to cited references that either directly address, or may be associated with, each topic. Some references can thus be found in several of the categories.

Topics	References
Functional involvement of TIC markers in TIC metabolism	[5,6,20,36,37,38,42,43,51,55]
Influence of growth and culture conditions on TIC-ness Levels/effects of hypoxia, nutrients or cytokines Tumor microenvironment On physiological models for in vitro studies on TICs	[1 *,^2^ *,^13^ *,^14^,^41^,^61^,^62^ *,^63^,^64^ *,^81^,^82^ *,^89^,^90^] [2 *,^13^ *,^48^,^61^,^62^ *,^63^,^82^,^83^,^84^,^85^ *,^88^,^92^] [2 *,^13^ *,^92^]
Tumor sample heterogeneity with regard to TICs Defining TICs, DTPs, MICs Expression signatures Overlap between bulk tumor cells/EMT and TICs	[14,15 *,^16^] [5,6,16,17,18,19,43,47,91] [6,12,13,16,52,91]
Roles of: Mitochondrial biogenesis/content Mitochondrial dynamics Fatty acid transporters and different lipids Lipid droplets	[33,34,40,66 *,^68^,^70^ *,^71^,^75^,^86^ *,^87^ *] [75,80] [45,46,48,50 *, 51,52,54,55,56,57] [16,48,49,50 *]

* Review; Abbreviations: DTP: drug-resistant persister; MIC: metastasis-initiating cells; EMT: epithelial–mesenchymal transition.

## Abbreviations

AMPK	adenosine monophosphate kinase
ALDH	aldehyde dehydrogenase
ASAH	acid ceramidase
ATP	adenosine triphosphate
ATX	autotaxin
bFGF	bovine fibroblast growth factor
Bmi1	polycomb complex protein-1 encoded by the *BMI1* gene (B-cell specific Moloney murine leukemia virus integration site 1)
CD	Cluster of differentiation
CPT	carnitine palmitoyl transferase
CSC	cancer stem cells
CSCN	cancer stem cell niche
DCA	dichloroacetate
DMEM	Dulbecco’s modified Eagle’s medium
DTP	drug-tolerant persisters
ECM	extracellular matrix
EGF	epidermal growth factor
EMT	epithelial–mesenchymal transition
EpCAM	epithelial cell adhesion molecule
ERRα	estrogen-related receptor α
ETC	electron transport chain
FA	fatty acid
FAO	fatty acid oxidation
FATP	fatty acid transporter protein
GLUT	glucose transporter
GTP	guanosine triphosphate
HIF	hypoxia-inducible factor
IL	interleukin
iPSC	induced pluripotent stem cell
ITGA	integrin alpha
JAK	Janus kinase
Klf	Krüppel-like factor
LD	lipid droplets
LDH	lactate dehydrogenase
LGR	leucine-rich repeat-containing G-protein coupled receptor
LPA	lysophosphatidic acid
MCL	induced myeloid leukemia cell differentiation protein
Mfn	mitofusin
MIC	metastasis-initiating
MPC	mitochondrial pyruvate carrier
miRNA	micro-RNA
MMP	mitochondrial membrane potential
MMTV	mouse mammary tumor virus
MUFA	mono-unsaturated fatty acid
NADPH	nicotine amide dinucleotide phosphate (reduced)
NFκB	nuclear factor kappa-light-chain enhancer of activated B cells
OCT	octamer-binding transcription factor
OXPHOS	oxidative phosphorylation
PDK	pyruvate dehydrogenase kinase
PEPCK	phosphoenolpyruvate carboxykinase
PFKFB	6-phosphofructo-2-kinase/fructose-2,6-biphosphatase
PGC	PPAR gamma co-activator
PPAR	peroxisome proliferator-activated receptor
PPP	pentose phosphate pathway
ROS	reactive oxygen species
SCD	stearoyl-CoA desaturase-1
shRNA	short hairpin RNA
SOX	sex-determining region Y-related high-mobility group box
SREBP	sterol regulatory element-binding protein
STAT	signal transducer and activator of transcription
TCA cycle	tricarboxylic acid cycle (citric acid cycle)
TFAM	mitochondrial transcription factor M
TIC	tumor-initiating cells
UQRCB	ubiquinol-cytochrome *c* reductase binding protein
VDAC	voltage-dependent anion channel
Wnt	wingless-related integration
YY	yin–yang
